# A Preliminary Study on the Safety of Elastography during Pregnancy: Hypoacusia, Anthropometry, and Apgar Score in Newborns

**DOI:** 10.3390/diagnostics10110967

**Published:** 2020-11-18

**Authors:** Paloma Massó, Juan Melchor, Guillermo Rus, Francisca Sonia Molina

**Affiliations:** 1 Department of Preventive Medicine, Hospital Universitario Virgen de las Nieves, 18014 Granada, Spain; pmasso@ugr.es; 2 Biomechanics Group (TEC-12), Instituto de Investigación Biosanitaria, ibs.GRANADA, 18012 Granada, Spain; grus@ugr.es (G.R.); fsoniamolina@gmail.com (F.S.M.); 3 Department of Statistics and Operations Research, University of Granada, 18071 Granada, Spain; 4 Excellence Research Unit “ModelingNature” MNat, University of Granada, 18071 Granada, Spain; 5 Department of Structural Mechanics, University of Granada, 18071 Granada, Spain; 6 Maternal-Fetal Medicine Unit, Department of Obstetrics and Gynaecology, Hospital Universitario San Cecilio, 18016 Granada, Spain

**Keywords:** elastography, ARFE, fetal safety, ultrasound safety, hypoacusia, anthropometric measurements, Apgar test score

## Abstract

Transient or acoustic radiation force elastography (ARFE) is becoming the most extended technology to assess cervical effacement, additionally to the Bishop test and conventional ultrasound. However, a debate on the fetal safety has been opened due to the high intensity focused beam emitted to produce shear waves. This work is aimed at providing preliminary data to assess clinical effects of fetal exposure. A follow-up study in newborns of 42 women exposed to ARFE during pregnancy was carried out to explore neonatal hypoacusia, Apgar test, and anthropometry. No hypoacusia cases attributable to ARFE were observed. The Apgar test at five minutes scored normally in all the newborns. Comparisons between anthropometric measurements showed no significant statistically differences. The results preclude to state the harmfulness nor the safety of ARFE. However, given the concern on the high level of energy and the potential risk of harmful bioeffects, larger studies are recommended.

## 1. Introduction

Quasi-static ultrasonic elastography techniques are increasing interest to assess the stiffness of cervical tissue during pregnancy [1,2,3,4,5]. Although static elastography was the first technique, ARFE promises higher reproducibility and objectivity. This technique is already provided as a mode of operation included in standard echography, but it also takes advantage of the phenomenon of the nonlinear radiation force [6] to generate a visible pulsed shear deformation through a sufficiently concentrated burst of compression waves. However, the high level of energy in the pulses emitted has not been considered in setting the current standard safety criteria: the mechanical index (MI) to assess cavitation effects, and thermal index (TI) related to temperature rise.

According to the US Food and Drug Administration (FDA), fetal exposure to general ultrasound is limited to a maximum spatial peak pulse average intensity of 720 mW/cm${}^{2}$ and a maximum mechanical index of 1.9 [7,8]. Both time averages indices are not suited to assess the magnitude of mechanical energy intensity in peaks concentrated during a given interval of microseconds per second according to the multi-frame elastography. There is no evidence on the potential harmful bioeffects of such energy peaks on sensitive structures, although therapeutic applications based on the histological effects of ultrasounds have been tested [9,10]. Since the mechanism of ultrasound in living tissue is not described in depth, phenomena as absorption, perfusion, cell membrane sonoporation or denaturalization, growth factor cell signaling and mechano-transduction or molecular effects, to name a few likely involved processes, should be further assessed.

The potential teratogenicity of elastography has been commented on in the last few years [11,12,13,14] and discussed by Preis and Rus [15] reported no histological changes in the placentas measured ex vivo by ARFE, but they could not probe the absence of other harmful bioeffects and suggested further studies. Tabaru et al. [16] were confident on the safety of applying high intensity energy in maternal examination by ARFE arguing that shear waves are not transmitted through amnion liquid. However, this assumption disregards burst compression effects of the ultrasonic peak, and the fact that the ARFE technique is not only applied to maternal examination but also to fetal diagnosis. In silico simulations of the ARFE thermal effects in bone tissue interfaces showed concentrated build-ups bordering safety thresholds [17,18]. In ARFE examination during pregnancy, cochlea and semicircular canals of the fetus may be considered sensitive tissues to the ultrasound energy peaks, given that these structures are immature and can be damaged by strong mechanical vibrations, in a similar way as several authors postulated for the neonatal brain [12,19]. This hypothesis gains strength by the report of pulsed diagnostic ultrasound observed to increase fetal activity during exposure [19,20].

Despite the fact that there is no available specific data on the safety of ARFE use for fetuses among the studies performed in pregnant women, SuperSonic Imagine (SSI) Aixplorer ultrasound system has the approval for in vivo use by the Europe Union’s Certificate (EC) and the US Food and Drug Administration (FDA). In accordance with current regulations, the use of ARFE is commonly supported by the argument that the thermal and mechanical indices of energy used are equal to or slightly higher than that employed, respectively, by Doppler and conventional B-mode imaging ultrasound technologies [21], both widely applied for fetal diagnosis in the clinical practice. The caution principle has guided the protocols of application of ARFE technology during pregnancy, specifically for fetal diagnosis, e.g., by minimizing the duration of exposure to reduce the risk [22]. However, to be consistent to ALARA (As Low As Reasonably Achievable) principle, which ensures that the total ultrasound energy is below a level at which bioeffects are produced while diagnostic information is achieved, there is a lack of basic and clinical research focused on identifying and measuring potential hazards of the ARFE imaging application in fetal medicine [23]. In this article, we present the results of a follow-up study in the cohort of children born to 42 asymptomatic women exposed to ARFE during pregnancy to assess neonatal hypoacusia, anthropometric measurements and Apgar test score.

## 2. Materials and Methods

### 2.1. Subjects

This work is a follow-up of a cross-sectional study in 42 women with uncomplicated pregnancy, between 6 to 41 weeks’ gestation (mean of 27.5), undergoing ARFE examination at San Cecilio University Hospital in Granada, Spain in 2012 [24]. Non-inclusion criteria were communication problems, prior cervical surgery (e.g., conization or cerclage), and premalignant or malignant histological changes of the cervix. The study was designed according to the Declaration of Helsinki and with the approval of the local ethical committee in human investigation (Comité de Ética en Investigación Humana de la Universidad de Granada and Comisión de Ética e Investigación Sanitaria del Hospital Universitario San Cecilio de Granada). All the subjects agreed by signing the patient information sheet and the written informed consent.

### 2.2. Reference Population

The global reference rates of hypoacusia were taken from the World Health Organization standards [25], as well as the reference anthropometric scores of weight, length, BMI, and cranial perimeter in newborns.

### 2.3. Acoustic Radiation Force Elastography

**Figure 1:**
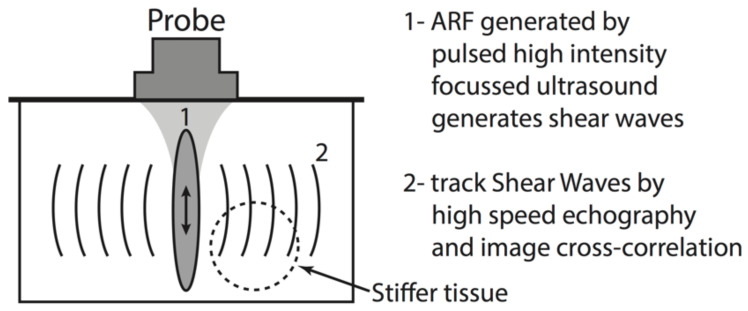
Schematic principle of ARFE.

Elastography was performed in the exposed group using an ARFE-based commercial Supersonic Shear Imaging device (SSI) and a 7-MHz conventional endocavitary ultrasonic probe (SE 12-3, Supersonic Imagine, Aix-en-Provence, France). This technique [26,27] consists on generating shear waves inside the tissue by acoustic radiation force by a focussed ultrasound beam (Figure 1) [6].

The ultrasound radiation force generates a broadband low frequency and high intensity pulsed shear wave (300–800 Hz), which propagates perpendicularly to the ultrasonic beam axis. Right after the shear waves generation, the system is then switched into an ultrafast ultrasound plane wave imaging mode (up to 20,000 frames/s) to capture the wave propagation and measure the shear wave velocity. From this shear wave velocity, it is possible to compute a shear elasticity map under the assumption of a purely elastic and incompressible medium. The endocavitary ultrasonic probe was set at 7 MHz (SE 12-3, Supersonic Imagine, Aix-en-Provence, France).

Prior to ARFE examination, the women were asked to empty their bladder and afterwards were placed in lithotomy position. The probe was situated in the anterior fornix of the vagina for a sagittal view of the cervix, aligned to the echogenic mucosa of the endocervical canal. Four measurements to ARFE were obtained in each subject by two operators to validate the interobserver reproducibility, and twice each operator to test the intraobserver repeatability. The measurements were located in four regions of interest (ROI): the cervix, the outer and upper lip, the inner and upper lip, the inner and lower lip, and the outer and lower lip. In each region, a 6 mm diameter circle was placed to display automatically a stiffness value (kPa), as well as the mean value and the standard deviation [24].

### 2.4. Outcomes

The outcomes of this study were a positive audiologic test for hypoacusia, newborn anthropometric measurements (weight, length, BMI, and cranial perimeter), and Apgar scores at 1 and 5 min at birth to assess vitality and health condition in the newborn. TORCH, syndromic, and hereditary causes of hypoacusia were excluded. Data were collected from medical records, and the individual reports of the results obtained from the universal screening program to prevent neonatal hypoacusia in Andalusia. The audiologic test consisted of the Transient Evoked Otoacoustic Emissions (TEOAEs) technique. Only in positive cases was a second step of automated Auditory Evoked Potentials test (aAEPT) performed.

### 2.5. Statistical Analysis

The risk of the ARFE fetal exposure for hypoacusia was estimated by calculating differences of prevalence between the study population and the reference population, considered as asymptotically large, through a contingency table and a chi-square statistic test. We calculated the mean, range, and SD for Apgar test at 1 and 5 min to compare with normal values. To assess the potential effects of fetal exposure to ARFE on newborn anthropometric measurements, comparisons of weight, length, BMI, and cranial perimeter at birth between the children of the study and the reference population were made by the Wilcoxon rank sum test with continuity correction, and conversions to Z-scores by gender. Comparison of anthropometric scores represented through boxplots was made by a normal distribution simulation.

The statistical analysis was carried out on R software version 3.3.1, with epiR version 0.9-79 package (R Core Team 2014) [28].

## 3. Results

### 3.1. Characteristics of Population and Apgar Test Score

A total of 42 children were born to the 41 women aged 17 to 43 years undergoing to ARFE examination (four measurements per subject). The characteristics of the mothers and newborns in the study are showed in Table 1. The mean of gestational age at delivery was 39.6 (33.3–41.9) weeks. A unique preterm delivery was presented (2.38%). The mean of gestational age at ARFE examination time was 27.5 (6–41) weeks [24]. The average of Apgar scores at 1-min of birth was 8.7 (4–10) and at 5-min was 9.7 (7–10). In five subjects of the sample, the results of the audiologic screening test were not registered in the medical records, so the parents were phoned to be asked if their child had hearing loss in the last years. Finally, outcome data on hypoacusia test was obtained in all subjects. There were missing data on cranial perimeter in six subjects (14.3%), weight in one subject (2.4%) and length in five subjects (11.9%), as the values were no legible or available in medical records. One twin baby who met the exclusion criteria of a syndromic cause of hypoacusia was ruled out of the analyses, as discussed later.

### 3.2. Outcomes on Hypoacusia

All of the newborns in our study had a negative result in the audiologic test, except one case affected with Prader–Willi-like syndrome, which was not considered a positive outcome. The comparison of the prevalence of diagnosis of hypoacusia in the sample of the study and the reference population is shown in Table 2. Nonetheless, medical records of this particular case and her mother were carefully reviewed to discard a teratogenous effect of ARFE technique, and a detailed description of the clinical context of this subject is summarized below. We calculated the Risk Etiologic Fraction in the exposed sample (REF) to estimate the proportion of hypoacusia attributable to the fact of being exposed to ARFE during fetal period. The negative value of −0.5% of hypoacusia among the exposed children was due to the limited size of the sample, as discussed later. Although no hypoacusia cases attributable to ARFE exposition were observed, the sample size provided no statistically significance (*p* = 0.538, Table 3).

The positive case for hypoacusia was reported as a late premature (36 weeks’ gestation, 1860 g) female newborn, intrauterine growth restriction from the 34 week’s gestation, and was the second born from a dichorionic-diamniotic twin pregnancy. The mother was a 35-year-old primiparous, with controlled gestational diabetes, and the pregnancy was obtained through in vitro fecundation. At the delivery time, the phenotype of the second newborn showed some dysmorphic features (fissures in the lobes of both pinnae, horizontal palpebral fissures, short philtrum, thin lips, cupid bow upper lip, and crossed feet fingers) and other anomalies, such as an asymmetry in cerebral ventricles, megacistern magna, and bilateral hydronephrosis, were detected through echography. At seven months of age, a CGH Cytoarray ISCA (60K) test confirmed a chromosome 6p16.3-q21 deletion of 12.48 Mb (cr6:100617272-113097226). Several audiological tests (TEOAEs and AEPT) were repeated at 2, 8, and 10 months of age (the last two during hospitalization to perform a tympanic paracentesis), showing positive results. The last audiologic tests (AEPT by air and bone and by Auditory Steady-State Response (ASSR) under general anaesthesia) at three years old detected a bilateral mild sensorineural hypoacusia of 30–40 db.

### 3.3. Outcomes on Anthropometry

**Figure 2:**
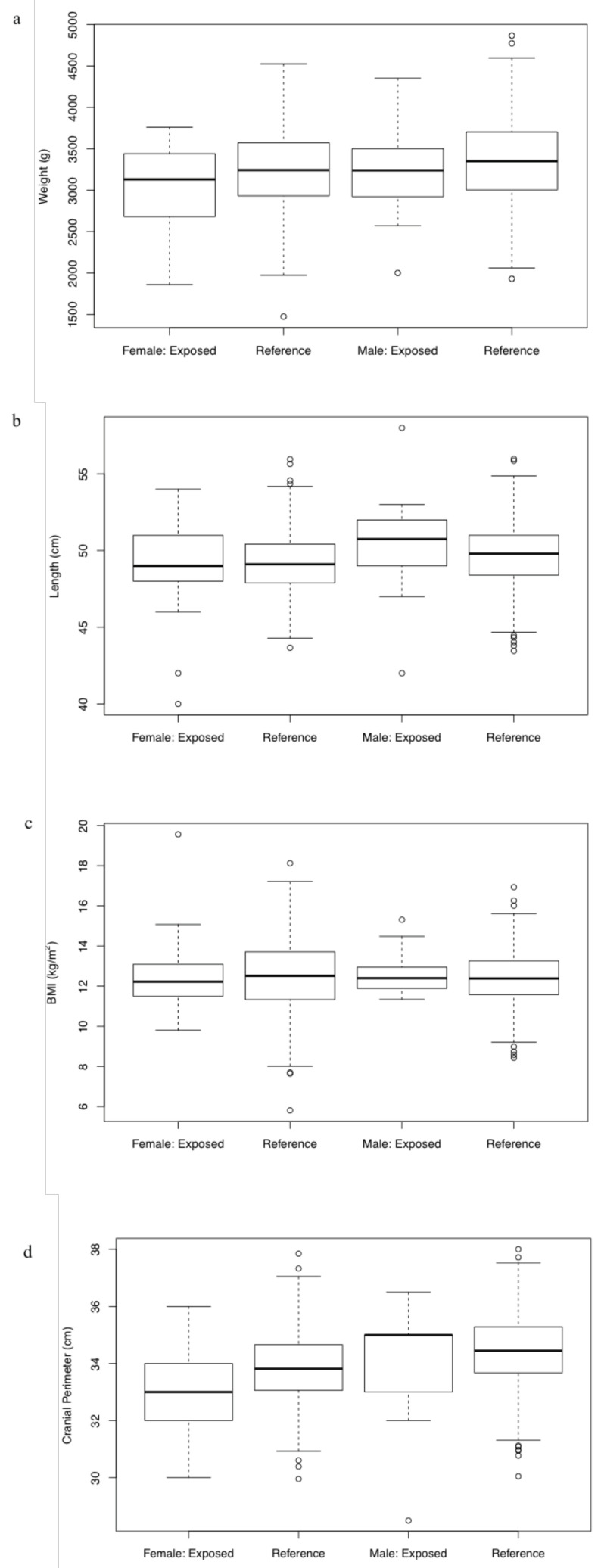
(**a**–**d**) Comparisons of anthropometric scores (weight, length, BMI, and cranial perimeter) between the exposed children and the reference WHO population.

Comparisons between the values of anthropometry in the sample of the study and the reference population did not demonstrate significant statistically differences on weight (*p* = 0.1233), length (*p* = 0.1582), BMI (*p* = 0.94860) and cranial perimeter (*p* = 0.06608) due to ARFE fetal exposure (Figure 2). Anthropometric Z-scores for length, weight, BMI, and cranial perimeter showed normal values (<−1 SD) both in females and males newborns in the study in relation to the gendered reference population. The mean of weight, BMI, and cranial perimeter in both sexes in the subjects of the study were below the mean in the reference population, whereas the mean of length was higher (<+1 SD). Therefore, percentiles of weight, BMI, and cranial perimeter were lower than percentiles of length ($P_{25-50}$ versus $P_{50-75}$) both in males and females of the study referred to the WHO population (Table 4).

## 4. Discussion

Although acoustic radiation force elastography complies with MI and TI, there are physical reasons why it may still be teratogenous. In fact, bio-effects due to cavitation and thermal interactions have not been sufficiently monitored in fetal imaging. In this study, we focused on the cochlea as a possible target organ of damage, as well as anthropometric measurements and Apgar scores at birth, through a review of the results obtained in the screening test for hypoacusia and physical examination in the newborns.

In our study, no positive outcome for hypoacusia meeting the selection criteria was founded. The only case for the audiologic test detected was excluded due to a phenotype Prader–Willi-like (PWL). This is related to a rare chromosomopathy which associates the dysplastic features observed in the newborn, as well as developmental delay, hypotonia, and obesity [29,30,31]. Occasionally, ear malformations (in this case, fissures in the lobes of both pinnae) are related to neurological defects as sensorineural retrocochlear hypoacusia [31,32], even when these dysplasias are just observed as an isolated feature [33,34]. On the other hand, the ARFE exposure in this subject happened at 34 weeks and five days of gestational age, so a teratogenous effect of the ARFE can be excluded since the organogenesis of the inner ear is almost completed at week 10 [35]. Hence, taking into account all the described clinical aspects of the positive case in our study, and that any more case of hypoacusia has been detected, we can consider the pathological finding attributable to the PWL syndrome. Nevertheless, the hypothesis of a traumatic cochlear or retrocochlear damage caused by the ARFE, simultaneously or not to a syndromic sensorineural hypoacusia, cannot be totally rejected. It should be noted that an improvement of hearing was observed in this child during clinical follow-up. Fortunately, this evolution is consistent to recent evidence on hearing recovery in preterm children in the long term [36].

The average of Apgar test score at 1-min was normal and it improved at 5-min, observing normal values in all the newborns (>7). On the other hand, the results of the study showed no statistically significant differences in anthropometric measurements between the newborns in the sample and the reference population. Nonetheless, the slightly lower scores of weight, BMI, and cranial perimeter ($P_{25-50}$) observed in the cohort of the study could be due to a potential bias in the sample. Despite the fact that women were recruited in their medical visits to control their uncomplicated pregnancies, it should be noted that the study was carried out in the Fetal Medicine Unit at the Hospital, where the prevalence of high risk pregnancy is higher compared to other clinical settings. Other limitations of the study are linked to the validity of the audiologic diagnostic techniques. Neither TEOAEs nor aAEPT reach 100% of sensitivity and specificity [37,38,39,40], therefore false negatives and false positives results could be included among outcome of hypoacusia in the sample. On the other hand, a monitoring the prevalence of neonatal hypoacusia in the Andalusian reference population would be required to calculate the positive predictive value (PPV) and the negative predictive value (NPV), as well as the proportion of syndromic and other types of hypoacusia causes among the positive cases in further research.

Currently, the uncertainty of ARFE bioeffects leads to recommending avoiding fetal exposure before 12 weeks of gestation to ensure that the organogenesis is sufficiently completed, as well as to minimize the duration examination in pregnancy.

## 5. Conclusions

The strength of this study is that it is a pioneer for addressing the safety of ARFE for human fetuses. Unfortunately, the limitation of the sample size underpowered results to conclude the harmfulness nor the safety of fetal exposure to ARFE. Some constraints of the follow-up are due to the design and methodological issues of the former study, which was performed four years before the debate on the safety of elastography was opened. A future study aimed at assessing teratogenic hypoacusia due to ARFE exposure will require a sample of 772 subjects to have 80% power for detecting statistically significant differences at a confidence level of 95%. Given the recent concerns about the high intensity of energy pulses required for ARFE imaging, further studies in fetal safety with a larger sample are needed.

## Figures and Tables

**Table 1:** Descriptive analysis of the characteristics of mothers and newborns in the study population.

	N %	Mean/Range	SD
*Mothers*			
Age (years)		32.2(17-42)	5.4
Delivery mode:			
Vaginal	29 (69.0)		
Cesarean section	13 (31.0)		
Gestational age at delivery (wk)		39.6 (33.3-41.9)	1.9
Gestational age at ARFE test (wk)		27.5 (6.0-41.0)	10.8
**Total**	**41**		
*Newborns*			
Sex:			
Female	25 (59.5)		
Male	17 (40.5)		
1-min Apgar score		8.7 (4-10)	1.0
5-min Apgar score		9.7 (7-10)	0.7
Preterm birth	1 (2.4)		
**Total**	**42**		

**Table 2:** Contingency table to compare the prevalence of diagnosis of hypoacusia in the sample of the study and the reference population. * A syndromic case was excluded, according to the criteria of the study.

	Hypoacusia %	Normal Audition *N*	Total *N*
ARFE exposed population	0 *	41	41
Reference population	0.5	-	-

**Table 3:** Risk Etiologic Fraction (REF) and Chi-square test estimates of effect of ARFE fetal exposure on hypoacusia in newborns

OR	REF %	$\chi^{2}$-Test	*p*-Value
0	−0.5	0.38	0.538

**Table 4:** Descriptive analysis of anthropometric scores at birth in the sample and comparison to the reference population.

Anthropometric Scores at Birth	Mean/ Range		SD		Z-Scores		WHO Percentile Range		*p*-Value
	F	M	F	M	F	M	F	M	
Weight (g)	3052.4 (1860–3760)	3278.8 (2000–4350)	503.8	588.6	−0.36	−0.11	25–50	25–50	0.1233
Length (cm)	49.1 (40–54)	50.5 (42–58)	3.1	3.6	0.16	0.18	50–75	50–75	0.1582
BMI (kg/m${}^{2}$)	12.6 (9.8–19.6)	12.7 (11.3–15.3)	2.0	1.2	−0.38	−0.64	25–50	25–50	0.94860
Cranial perimeter (cm)	33.1 (30–36)	34.2 (28.5–36.5)	1.5	2.1	−0.51	−0.14	25–50	25–50	0.06608

## References

[1] Khalil M.R., Thorsen P., Uldbjerg N. (2013). Cervical ultrasound elastography may hold potential to predict risk of preterm birth. Dan. Med..

[2] Köbbing K., Fruscalzo A., Hammer K., Möllers M., Falkenberg M., Kwiecien R., Klockenbusch W., Schmitz R. (2014). Quantitative elastography of the uterine cervix as a predictor of preterm delivery. J. Perinatol..

[3] Swiatkowska-Freund M., Preis K. (2011). Elastography of the uterine cervix: Implications for success of induction of labor. Ultrasound Obstet. Gynecol..

[4] Pereira S., Frick A.P., Poon L.C., Zamprakou A., Nicolaides K.H. (2014). Successful induction of labor: Prediction by preinduction cervical length, angle of progression and cervical elastography. Ultrasound Obstet. Gynecol..

[5] Wozniak S., Czuczwar P., Szkodziak P., Milart P., Wozniakowska E., Paszkowski T. (2014). Elastography in predicting preterm delivery in asymptomatic, low-risk women: A prospective observational study. BMC Pregnancy Childbirth.

[6] Rus Carlborg G. (2014). Nature of acoustic nonlinear radiation stress. Appl. Phys. Lett..

[7] Frigoletto F., Auerbach R., Brickler A. (1984). Consensus conference: The use of diagnostic ultrasound imaging during pregnancy. JAMA.

[8] Nyborg W.L. (2001). Biological effects of ultrasound: Development of safety guidelines. Part II: General review. Ultrasound Med. Biol..

[9] Mundi R., Petis S., Kaloty R., Shetty V., Bhandari M. (2009). Low-intensity pulsed ultrasound: Fracture healing. Indian J. Orthop..

[10] Padilla F., Puts R., Vico L., Raum K. (2014). Stimulation of bone repair with ultrasound: A review of the possible mechanic effects. Ultrasonics.

[11] Massó P., Rus G., Molina F. (2017). Safety of elastography in fetal medicine: Preliminary study on hypoacusis. Ultrasound Obstet. Gynecol..

[12] Li C., Zhang C., Li J., Cao X., Song D. (2016). An experimental study of the potential biological effects associated with 2D shear wave elastography on the neonatal brain. Ultrasound Med. Biol..

[13] Kılıç F., Kayadibi Y., Yüksel M.A., Adaletli İ., Ustabaşıoğlu F.E., Öncül M., Madazlı R., Yılmaz M.H., Mihmanlı İ., Kantarcı F. (2015). Shear wave elastography of placenta: In vivo quantitation of placental elasticity in preeclampsia. Diagn. Interv. Radiol..

[14] Carlson L.C., Feltovich H., Palmeri M.L., Dahl J.J., Munoz del Rio A., Hall T.J. (2014). Estimation of shear wave speed in the human uterine cervix. Ultrasound Obstet. Gynecol..

[15] Sugitani M., Fujita Y., Yumoto Y., Fukushima K., Takeuchi T., Shimokawa M., Kato K. (2013). A new method for measurement of placental elasticity: Acoustic radiation force impulse imaging. Placenta.

[16] Tabaru M., Yoshikawa H., Azuma T., Asami R., Hashiba K. (2012). Experimental study on temperature rise of acoustic radiation force elastography. J. Med. Ultrason..

[17] Palmeri M.L., Frinkley K.D., Nightingale K.R. (2004). Experimental studies of the thermal effects associated with radiation force imaging of soft tissue. Ultrason. Imaging.

[18] Liu Y., Herman B.A., Soneson J.E., Harris G.R. (2014). Thermal safety simulations of transient temperature rise during acoustic radiation force-based ultrasound elastography. Ultrasound Med. Biol..

[19] Fatemi M., Alizad A., Greenleaf J.F. (2005). Characteristics of the audio sound generated by ultrasound imaging systems. J. Acoust. Soc. Am..

[20] Stratmeyer M.E., Greenleaf J.F., Dalecki D., Salvesen K.A. (2008). Fetal ultrasound: Mechanical effects. J. Ultrasound Med..

[21] Muller M., Aït-Belkacem D., Hessabi M., Gennisson J.L., Grangé G., Goffinet F., Lecarpentier E., Cabrol D., Tanter M., Tsatsaris V. (2015). Assessment of the cervix in pregnant women using shear wave elastography: A feasibility study. Ultrasound Med. Biol..

[22] Diguisto C., Simon E.G., Callé S., Ternifi R., Remeniéras J.P., Hervé P., Perrotin F. (2017). Ultrasonic elastography exploration of the foetal brain: A case of atypical choroid plexus papilloma. J. Obstet. Gynaecol..

[23] Orenstein B. (2011). The ALARA principle and sonography. Radiol. Today.

[24] Peralta L., Molina F.S., Melchor J., Gómez L.F., Massó P., Florido J., Rus G. (2017). Transient elastography to assess the cervical ripening during pregnancy: A preliminary study. Ultraschall Med..

[25] WHO International (2019). WHO Global Estimates on Prevalence of Hearing Loss. Mortality and Burden of Diseases and Prevention of Blindness and Deafness, 2012.

[26] Bercoff J., Tanter M., Fink M. (2004). Sonic boom in soft materials: The elastic Cerenkov effect. Appl. Phys. Lett..

[27] Bercoff J., Tanter M., Fink M. (2004). Supersonic shear imaging: A new technique for soft tissue elasticity mapping. IEEE Trans. Ultrason. Ferroelectr. Freq. Control.

[28] R Core Team (2012). R: A Language and Environment for Statistical Computing.

[29] Spreiz A., Müller D., Zotter S., Albrecht U., Baumann M., Fauth C., Erdel M., Zschocke J., Utermann G., Kotzot D. (2010). Phenotypic variability of a deletion and duplication 6q16. 1? q21 due to a paternal balanced ins (7; 6)(p15; q16. 1q21). Am. J. Med. Genet. Part.

[30] Bonaglia M.C., Ciccone R., Gimelli G., Gimelli S., Marelli S., Verheij J., Giorda R., Grasso R., Borgatti R., Pagone F. (2008). Detailed phenotype–genotype study in five patients with chromosome 6q16 deletion: Narrowing the critical region for Prader–Willi-like phenotype. Eur. J. Hum. Genet..

[31] Iafrate A.J., Feuk L., Rivera M.N., Listewnik M.L., Donahoe P.K., Qi Y., Scherer S.W., Lee C. (2004). Detection of large-scale variation in the human genome. Nat. Genet..

[32] Rosenfeld J.A., Amrom D., Andermann E., Andermann F., Veilleux M., Curry C., Fisher J., Deputy S., Aylsworth A.S., Powell C.M. (2012). Genotype–phenotype correlation in interstitial 6q deletions: A report of 12 new cases. Neurogenetics.

[33] (2007). Joint Committee on Infant Hearing. Year 2007 position statement: Principles and guidelines for early hearing detection and intervention programs. Am. Acad. Pediatr..

[34] Trinidad-Ramos G., de Aguilar V.A., Jaudenes-Casaubón C., Núñez-Batalla F., Sequí-Canet J.M. (2010). Recomendaciones de la Comisión para la Detección Precoz de la Hipoacusia (CODEPEH) para 2010. Acta Otorrinolaringol. Ógica Esp..

[35] Sadler T.W. (2006). Langman’s Medical Embryology.

[36] Yang H., Sung C., Shin D., Cho Y., Jang C., Cho H.H. (2017). Newborn hearing screening in prematurity: Fate of screening failures and auditory maturation. Clin. Otolaryngol..

[37] Cubillana-Herrero J.D., Pelegrín-Hernández J.P., Soler-Valcarcel A., Mínguez-Merlos N., Cubillana-Martínez M.J., Barrios Á.N., Medina-Banegas A., Hernandez J.A.F. (2016). The assessment of the Newborn Hearing Screening Program in the Region of Murcia from 2004 to 2012. Int. J. Pediatr. Otorhinolaryngol..

[38] Vohr B.R., Carty L.M., Moore P.E., Letourneau K. (1998). The Rhode Island hearing assessment program: Experience with statewide hearing screening (1993–1996). J. Pediatr..

[39] Stewart D.L., Mehl A., Hall J.W., Thomson V., Carroll M., Hamlett J. (2000). Universal newborn hearing screening with automated auditory brainstem response: A multisite investigation. J. Perinatol..

[40] Norton S.J., Gorga M.P., Widen J.E., Folsom R.C., Sininger Y., Cone-Wesson B., Vohr B.R., Mascher K., Fletcher K. (2000). Identification of neonatal hearing impairment: Evaluation of transient evoked otoacoustic emission, distortion product otoacoustic emission, and auditory brain stem response test performance. Ear Hear..

